# Short Method of Procuring Plaster Impressions for Partial Sets

**Published:** 1861-02

**Authors:** D. B. Ingalls


Short Method of Procuring Plaster Impressions for Partial Sets.—By *D. B. Ingalls.*—Take an impression in wax in the usual way, using an impression cup large enough to take in the remaining teeth, as far back as is necessary, and a little more of the palatine arch than you wish your plate to cover. Then with a heated knife cut out a cavity in the wax impression the size you intend to make your plate, trim the wax, in making the cavity, as near the depressions made by the remaining teeth as necessary, to get a perfect plaster impression, and then the remaining wax will be a sufficient safeguard to keep the plaster from flowing around the teeth.

When the wax impression is prepared to suit, (and the preparation may include the "wooden fibres," etc., if necessary,) fill the cavity made in it, with this plaster, replace it in the mouth, and allow it to remain until the plaster becomes quite hard.

If you have done all with care, you will have no difficulty in removing the impression from the mouth, and can hardly fail of having an accurate impression.—*Dental Cosmos.*

